# Activation of the nuclear receptor PPARδ is neuroprotective in a transgenic mouse model of Alzheimer’s disease through inhibition of inflammation

**DOI:** 10.1186/s12974-014-0229-9

**Published:** 2015-01-16

**Authors:** Tarja Malm, Monica Mariani, Lauren J Donovan, Lee Neilson, Gary E Landreth

**Affiliations:** Department of Neurosciences, Alzheimer Research Laboratory, School of Medicine, Case Western Reserve University, Cleveland, OH 44106 USA; Department of Neurobiology, A.I.Virtanen Institute for Molecular Sciences, University of Eastern Finland, P.O. Box 1627, 70211 Kuopio, Finland

**Keywords:** Alzheimer’s disease, Microglia, Phagocytosis, Aβ, Neurodegeneration, Nuclear receptor, Inflammation, PPARδ

## Abstract

**Background:**

Alzheimer’s disease (AD) is a multifactorial disorder associated with the accumulation of soluble forms of beta-amyloid (Aβ) and its subsequent deposition into plaques. One of the major contributors to neuronal death is chronic and uncontrolled inflammatory activation of microglial cells around the plaques and their secretion of neurotoxic molecules. A shift in microglial activation towards a phagocytic phenotype has been proposed to confer benefit in models of AD. Peroxisome proliferator activator receptor δ (PPARδ) is a transcription factor with potent anti-inflammatory activation properties and PPARδ agonism leads to reduction in brain Aβ levels in 5XFAD mice. This study was carried out to elucidate the involvement of microglial activation in the PPARδ-mediated reduction of Aβ burden and subsequent outcome to neuronal survival in a 5XFAD mouse model of AD.

**Methods:**

5XFAD mice were orally treated with the PPARδ agonist GW0742 for 2 weeks. The brain Aβ load, glial activation, and neuronal survival were assessed by immunohistochemistry and quantitative PCR. In addition, the ability of GW0742 to prevent direct neuronal death as well as inflammation-induced neuron death was analyzed *in vitro*.

**Results:**

Our results show for the first time that a short treatment period of 5XFAD mice was effective in reducing the parenchymal Aβ load without affecting the levels of intraneuronal Aβ. This was concomitant with a decrease in overall microglial activation and reduction in proinflammatory mediators. Instead, microglial immunoreactivity around Aβ deposits was increased. Importantly, the reduction in the proinflammatory milieu elicited by GW0742 treatment resulted in attenuation of neuronal loss *in vivo* in the subiculum of 5XFAD mice. In addition, whereas GW0742 failed to protect primary neurons against glutamate-induced cell death, it prevented inflammation-induced neuronal death in microglia-neuron co-cultures *in vitro*.

**Conclusions:**

This study demonstrates that GW0742 treatment has a prominent anti-inflammatory effect in 5XFAD mice and suggests that PPARδ agonists may have therapeutic utility in treating AD.

## Introduction

Alzheimer’s disease (AD) is a severe multifactorial disorder leading to progressive dementia and eventually death. One of the major hallmarks of AD is the accumulation of soluble beta-amyloid (Aβ) peptides within the brain and the deposition of fibrillar forms of Aβ extracellularly [1]. The accumulation of fibrillar Aβ-containing plaques leads to the proinflammatory activation of microglia and astrocytes that surround the deposits. The ineffective clearance of the fibrillar Aβ deposits results in their chronic production of cytotoxic factors that act to exacerbate AD-like pathology and neuronal death [2,3].

Peroxisome proliferator-activated receptors (PPARs) are ligand-activated transcription factors that regulate cellular metabolism by binding to sequence-specific DNA elements. There are 3 PPAR isoforms, α, β/δ (hereafter referred to as PPARδ) and γ. In general, PPARs are lipid sensors and principally regulate fatty acid and cholesterol metabolism [4,5]. Importantly, PPARs act to regulate inflammatory processes in microglia and macrophages, suppressing the elaboration of cytokines and other inflammatory mediators and promoting tissue repair and phagocytosis [6,7]. PPARγ has been intensively studied in mouse models of central nervous system (CNS) diseases, including AD, in which its activation has been shown to lead to improvement in learning and memory and concomitant amelioration of AD-like pathology [8-11]. However, PPARδ is far less studied in models of brain diseases. PPARδ agonists have been shown to reduce the production of inflammatory mediators [12] especially in peripheral immune cells. PPARδ ligands have been shown to be neuroprotective in *in vivo* models of Parkinson’s disease (PD) [13,14], brain ischemia [14,15], spinal cord injury [16] and in streptozotocin-induced experimental diabetes [17]. Thus far, only a single study has addressed the effects of PPARδ activation in a mouse model of AD, showing that 1-month treatment of 5XFAD mice with PPARδ agonist GW0742 led to reduction in brain Aβ burden, reduced astrocytic activation and increased expression of Aβ-degrading enzymes [18]. Since the 5XFAD mice exhibit age-related neuronal degeneration in specific brain areas, we wished to dissect the protective effect PPARδ activation in 5XFAD mice in more detail, focusing especially on inflammation and AD-related neuronal death. Here we show that a 2-week oral treatment of 5XFAD mice with GW0742 reduced the brain Aβ load and microglial activation without affecting the number of neurons containing intracellular Aβ/amyloid precursor protein (APP). Importantly, we show for the first time that the treatment attenuated the degeneration of neurons in the subiculum of the 5XFAD mice. GW0742 was effective in preventing lipopolysaccharide (LPS)-induced increase in inflammatory mediators in primary microglia *in vitro*. Whereas GW0742 alone failed to prevent neuron loss against glutamate exposure, it significantly increased neuronal survival in inflammation-induced neuron death *in vitro*. Our data demonstrate that GW0742 is a powerful anti-inflammatory agent with neuroprotective properties and PPARδ agonism could be considered as a potential AD therapy.

## Materials and methods

### Animals and drug treatment

5XFAD male mice, originally described by Oakley *et al*., were a gift from Dr. Robert Vassar (Northwestern University) and B6SJL/F1 females were purchased from Jackson Laboratories (Bar Harbor, ME, USA). A total of 32 mice, both males and females were used in this study. The mice were randomized into study groups: wild type (WT) vehicle- treated: 9 female and 5 male mice; transgenic (TG) vehicle-treated: 4 female and 5 male mice and TG GW0742-treated: 5 female and 4 male mice. GW0742 was provided by GlaxoSmithKline (Research Triangle, NC, USA). GW0742 was given by oral gavage at the dose of 30 mg/kg daily as a water suspension for 2 weeks starting at the age of 4.5 months. Vehicle-treated mice received water only. The animals were sacrificed at the end of the treatment period 6 hours after the last dose of GW0742. All animal experimentation was done according to the Case Western Reserve University Institutional Animal Care and Use Committee guidelines.

#### Immunohistochemistry

At the end of the treatment period the mice were anesthetized with Avertin and transcardially perfused with 0.01 M, PBS pH 7.4. Brains were removed, and the right hemisphere was immersion fixed with 4% PFA in 0.1 M phosphate buffer (PB, pH 7.4) over night at 4°C. Thereafter, the brains were cryoprotected with 10% sucrose for 24 hours following incubation in 30% sucrose for 48 hours after which the brains were frozen and cut in serial 10-μM sagittal sections. The sections were incubated with antibodies to glial fibrillary acidic protein (GFAP; 1:500 dilution, Dako, Carpinteria, CA, USA), ionized calcium binding adaptor molecule 1 (Iba-1; 1:200 dilution, Wako Chemicals, Richmond, VA, USA), NeuN (Aves Labs Inc, Tigard, Oregon, USA), complement component 3 (C3) and C1qa (1:1,000 dilution, both from Novus Biologicals, Littleton, CO, USA,), and 6E10 (BioLegend, Dedham, MA, USA) followed by incubation with appropriate Alexafluor 488 or 546 conjugated secondary antibodies (Molecular Probes/Life Technologies, Grand Island, NY, USA).

Images of the hippocampi were taken from 3 sections per animal, approximately 1,200 μM apart and spanning the hippocampi. Immunoreactivity was quantified by using ImagePro Premium (Media Cybernetics, Rockville, MD, USA) blinded to the study groups and presented as percentage of positively-stained area in the hippocampi. NeuN positive neurons were counted in the subiculum region of the hippocampi in two sections from each animal. The cell count data weres confirmed by quantifying the percentage of NeuN immunoreactive area in the subiculum area of the hippocampi. Cortical 6E10 immunoreactive neuronal bodies were counted and cortical 6E10 immunoreactivity was quantified from a total of 3 separate images taken from layer V in the cortex. Intensity of the intraneuronal 6E10 immunoreactivity was quantified using ImageJ by outlining 10 to 14 individual 6E10 immunopositive neurons from 3 separate images of cortical layer V taken from 3 consecutive sections.

### Primary cortical neuronal cultures

Primary neurons were cultivated as described previously [19]. Briefly, cortices of embryonic day 15 C57BL/6J pups were dissected and freed from their meninges. After dissociation with 0.025% (w/v) trypsin in Krebs buffer (0.126 M NaCl, 2.5 mM KCl, 25 mM NaHCO_3_, 1.2 mM NaH_2_PO_4_, 1.2 mM MgCl_2_, 2.5 mM CaCl_2_, pH 7.4) for 20 minutes at 37°C the tissues were treated with 0.008% w/v DNaseI and 0.026% w/v trypsin inhibitor (Sigma, St. Louis, MO, USA) and centrifuged at 256 × g for 3 minutes. The cell pellet was resuspended in 3 ml of DNaseI/SBT1 (Sigma, St. Louis, MO, USA) in Krebs solution and gently triturated through a blunt-ended glass pipet. Seven milliliters of additional Krebs buffer were added, the cell suspension centrifuged at 256 × g for 3 minutes and the cells were resuspended in Neurobasal Medium (Gibco/Life Technologies, Grand Island, NY, USA) supplemented with 0.2 mM L-glutamine (Gibco, Grand Island, NY, USA), 0.01 mg/ml gentamicin (Sigma, St. Louis, MO, USA) and B27 Supplement (Gibco, Grand Island, NY, USA), filtered through a 200 μM nylon mesh filter and counted using a hemocytometer. Primary cortical neurons were plated onto poly-D-lysine (50 μg/ml in water) and laminin (5 μg/ml water; Sigma, St. Louis, MO, USA) 24-well plates at the density of 200,000 cells per well. After 5 to 6 days *in vitro* the cells were pre-exposed to 1 μM GW0742 for 6 hours followed by exposure to 500 μM glutamate in the presence of 1 μM GW0742 for 24 hours. Cell viability was measured by MTT assay. To assess the effect of 1 μM GW0742 in neuronal viability, the cells were exposed to 1 μM GW0742 alone.

### Primary microglia cultures

Primary microglia were cultivated as described previously by using mild trypsinization [11]. Briefly, P0-P3 C57BL/6J mouse pups were decapitated, the brains removed, rinsed with PBS containing 1 g/l glucose, mechanically dissociated and digested with 0.5% trypsin-EDTA for 20 minutes at 37°C. Thereafter, the tissue homogenate was resuspended in DMEM/F12 media (Gibco, Grand Island, NY, USA) containing 10% heat-inactivated FBS (Gibco, Grand Island, NY, USA) and 1% penicillin-streptomycin. After trituration the cell suspension was plated onto 150 mm culture dishes for 20 to 22 days at 37°C and 5% CO_2_. After the plates were confluent, astrocytes were removed by incubating the plates with 0.25% trypsin in Hank's Balanced Salt Solution (HBSS) diluted in 1:4 serum-free DMEM/F12 for 30 minutes to 1 hour at 37°C. After washing the plates with PBS, microglia attached on the plates were removed by trypsinization with 0.25% trypsin in PBS. The action of trypsin was stopped with DMEM/F12 media/10% heat-inactivated FBS, cells centrifuged and plated for subsequent studies. To analyze the effect of GW0742 on primary microglial viability, microglia cultures were exposed to 1 μM GW0742 for 24 hours and cell survival was analyzed by MTT assay.

### Primary neuron-microglia co-cultures

Primary neuron-microglia co-cultures were prepared as described by Gresa-Arribas *et al*. [20]. Briefly, primary cortical neurons were plated onto 24-well plate. At 5 to 6 days *in vitro* primary microglia were isolated and plated on the top of neurons in Neurobasal Medium (Gibco, Grand Island, NY, USA) supplemented with 0.2 mM L-glutamine (Gibco, Grand Island, NY, USA), 0.01 mg/ml gentamicin (Sigma, St. Louis, MO, USA) and B27 Supplement (Gibco, Grand Island, NY, USA) at the density of 1:2 (100,000 microglia per 200,000 neurons). The next day the co-cultures were exposed to 1 μM GW0742 for 6 hours after which they were exposed to 100 ng/ml LPS and 30 ng/ml interferon (IFN)γ (Preprotech, Rocky Hill, NJ, USA) for 48 hours. The cells were rinsed with PBS (pH 7.4), fixed with 4% PFA for 20 minutes, permeabilized with 0.2% Triton-x in PBS for 10 minutes and incubated with an anti-microtubule-associated protein 2 (MAP-2) antibody (Sigma, St. Louis, MO, USA) in 5% NGS following incubation with Alexa-488 conjugated secondary antibody (Molecular Probes, Eugene, OR, USA). Neuronal viability was evaluated by quantifying the extent of MAP-2 immunoreactivity in the microglia-neuron co-cultures. MAP-2 immunoreactivity reveals any alterations both in the dendritic compartment and the cell soma and is frequently used to assess neuronal integrity and viability in co-culture systems [20].

### Tissue dissection

At the time of sacrifice, the animals were terminally anesthetized with Avertin and perfused with PBS. Brains were removed and cortices dissected out. Hemibrains were homogenized in 800 μl of tissue homogenization buffer (250 mM sucrose, 20 mM Tris, 1 mM ethylenediaminetetraacetic acid (EDTA), 1 mM ethylene glycol tetraacetic acid (EGTA) in diethylpyrocarbonate-treated water) containing Protease Inhibitor Cocktail (1:100, Sigma, St. Louis, MO, USA). The homogenates were centrifuged at 5,000 × g for 10 minutes at 4°C and supernatants stored at −80°C and used for Western blot analysis.

### RT-PCR

For RT-PCR, primary microglia were plated at the density of 1 × 10^6^ cells per well and stimulated in serum-free DMEM/12 (Gibco, Grand Island, NY, USA) for 24 hours prior to stimulation with 1 μM GW0742 for 24 hours followed by 10 ng/ml LPS (Sigma, St. Louis, MO, USA) together with the GW0742 for 24 hours. Thereafter, the plates were washed with PBS and mRNA isolated using RNeasy Mini kit (Qiagen, Valencia, CA, USA) according to the manufacturer’s instructions.

Cortical brain samples were homogenized in homogenization buffer and an equivalent amount of RNABee (TelTest Inc, Friendwood, TX, USA) was added to the samples. Thereafter, 0.2 ml of chloroform (Sigma, St. Louis, MO, USA) were added, the samples centrifuged for 15 minutes at 13,000 × g at 4°C, equal amount of 70% ethanol was added to the aqueous layer followed by mRNA isolation using RNease Mini kit (Qiagen, Valencia, CA, USA). mRNA concentration and purity was determined using a NanoDrop 2000 (Thermo Scientific, Hudson, NH, USA). Equivalent amounts of mRNA were reverse transcribed using a QuantiTect Reverse Transcription kit (Qiagen, Valencia, CA, USA) according to the manufacturer’s instructions. The procedure included elimination of genomic DNA. The cDNA was preamplified for 14 cycles using a TagMan PreAmp Master Mix for select primer sets (Applied Biosystems/Life Technologies, Foster City, CA, USA). Quantitive PCR was performed with the StepOne Plus Real Time PCR system (Applied Biosystems, Foster City, CA, USA) for 40 cycles. Analysis of gene expression was performed using the comparative C_t_ method (ΔΔC_T_) where the threshold cycle for the target genes was normalized to glyceraldehyde 3-phosphate dehydrogenase (GAPDH) and rRNA internal housekeeping gene controls (ΔC_T_). The mRNA expression was presented as fold change and statistical analyses were performed on ΔC_T_ ± SEM for each target gene as described earlier [21].

### Western blotting

Protein concentration of the brain lysates was determined by BCA (Pierce, Rockford, IL, USA). Equal amounts of protein were run on Bis-Tris 4 to 12% gels (Life Technologies, Foster City, CA, USA). The following antibodies were used: anti-actin (Santa Cruz Biotechnology, Dallas, TX, USA); anti-apolipoprotein E (ApoE) (Santa Cruz Biotechnology, Dallas, TX, USA); anti-β-actin (Santa Cruz Biotechnology, Dallas, TX, USA); anti-ATP-binding cassette transporter A1 (Abca1; Novus Biologicals, Littleton, CO, USA) and G1 (Abcg1; Novus Biologicals, Littleton, CO, USA) followed by incubation with horseradish peroxidase (HRP)-conjugated secondary antibodies.

## Results

### Treatment of 5XFAD mice with GW0742 resulted in significant decrease in brain 6E10 immunoreactivity

**Figure 1:**
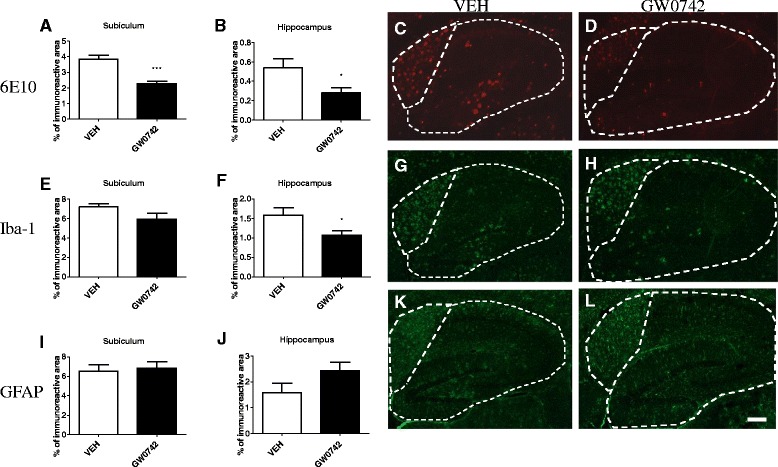
**GW0742 induced a significant decrease in brain 6E10 immunoreactivity.** Two-week treatment of 4.5-month-old 5XFAD mice with the PPARδ agonist GW0742 significantly reduced the 6E10 immunoreactivity in the subiculum **(A)** and hippocampus **(B)**. Figures **(C)** and **(D)** depict representative images of the 6E10 immunoreactivity in the hippocampal areas of vehicle and GW0742-treated mice, respectively. GW0742 failed to reduce Iba-1 immunoreactivity in the subiculum area of the hippocampi **(E)** but induced a significant reduction in microgliosis as measured by Iba-1 immunoreactivity in the hippocampus **(F)**. Figures **(G)** and **(H)** are representative images of Iba-1 immunoreactivity in hippocampal area in vehicle**- (G)** and GW0742- **(H)** treated mice. GW0742 treatment did not alter astrogliosis as measured by glial fibrillary acidic protein (GFAP) immunoreactivity in subiculum **(I)** or hippocampus **(J)**. Figures **(K)** and **(L)** are representative images of GFAP immunoreactivity in vehicle- **(K)** and GW0742- **(L)** treated mice. The dotted lines outline the quantified areas of the hippocampi and the subiculum. Results are presented as mean ± SEM. VEH = vehicle- and GW0742 = GW0742-treated mice.*P < 0.05 and ***P < 0.001 as analyzed by Student’s *t*-test. n = 7 in vehicle- and n = 9 in GW0742-treated group. Scale bar = 400 μm.

Two-week treatment of 4.5-month-old 5XFAD mice led to significant decrease in the 6E10 immunoreactivity in the subiculum and hippocampi (Figure 1A,B). It should be noted that in the 5XFAD model the 6E10-reactive species include full-length APP, C-terminal fragments (CTFs) and a diverse range of smaller and modified Aβ peptides [22-24]. Quantification of Iba-1 immunoreactivity revealed significant reduction of Iba-1 immunoreactivity in the hippocampus (Figure 1F) and we observed a trend toward lower levels in the subiculum that did not reach significance (Figure 1E). Astrocytic activation, as analyzed by quantification of GFAP immunoreactivity in the subiculum (Figure 1I) and hippocampus (Figure 1J) was not significantly altered by GW0742 treatment.

### Treatment with GW0742 increased abundance of microglia associated with 6E10 positive deposits

**Figure 2:**
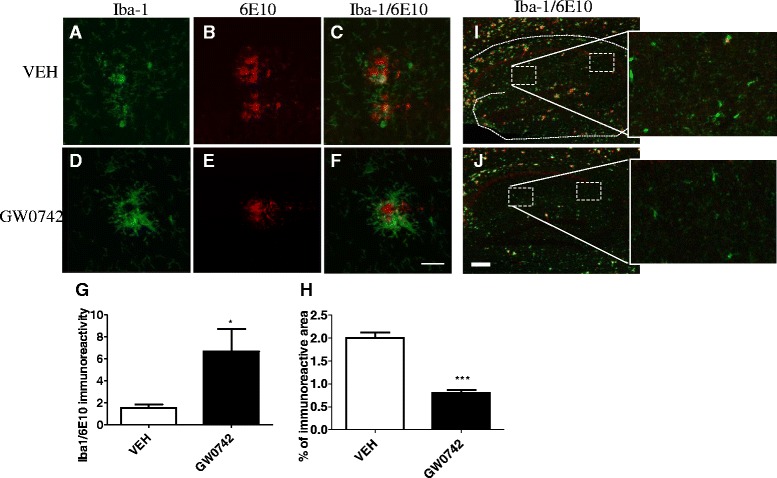
**GW0742 treatment increased the ratio of microglia/A** **β immunoreactivity.** Figure **(A)** shows a typical confocal image of microglia surrounding 6E10 immunopositive extracellular Aβ deposit **(B)** in vehicle-treated mice. Figure **(C)** is the overlay of **(A)** and **(B)**. Figure **(D)** shows a typical example of Iba-1 positive microglia in GW742-treated mice surrounding the Aβ deposits **(E)**. Figure **(F)** is the overlay of **(D)** and **(E)**. Scale bar = 100 μm. GW0742 treatment increased the ratio of Iba-1/6E10 immunoreactivity **(G)** and decreased Iba-1 immunoreactivity in areas devoid of 6E10 immunoreactivity **(H)**. Figures **(I)** and **(J)** with high magnification insets depict the quantified Iba-1 immunoreactive areas outside the areas of 6E10 immunoreactivity in vehicle **(I)** and GW0742 treated **(J)** mice. Scale bar = 400 μm. Results are presented as mean ± SEM. VEH = vehicle- and GW0742 = GW0742-treated mice. *P < 0.05 as analyzed by Student’s *t*-test. n = 7 in vehicle- and n = 9 in GW0742-treated group.

GW0742-treated animals had increased association of Iba-1 positive microglia surrounding the 6E10 positive Aβ deposits (Figure 2). The calculated ratio of Iba-1 and 6E10 immunoreactivity in GW0742-treated mice was significantly higher compared to vehicle- treated mice (Figure 2G). Moreover, quantification of Iba-1 immunoreactivity in the hippocampi in the areas between the 6E10 immunopositive plaques, and devoid of any 6E10 immunoreactivity revealed significantly reduced levels of Iba-1 staining in GW0742-treated animals (Figure 2H). The high magnification insets in the Figure 2I and J show the areas quantified and example images of the Iba-1 immunoreactivity in areas outside the 6E10 immunoreactivity in vehicle- and GW0742-treated mice, respectively. These data indicate that PPARδ activation results in recruitment of microglia to amyloid deposits, coincident with the clearance of the plaques. The overall reduction in Iba-1 positive microglia follows from clearance of plaques from the brain.

### GW0742 treatment did not reduce the number of neurons with intraneuronal APP/Aβ

**Figure 3:**
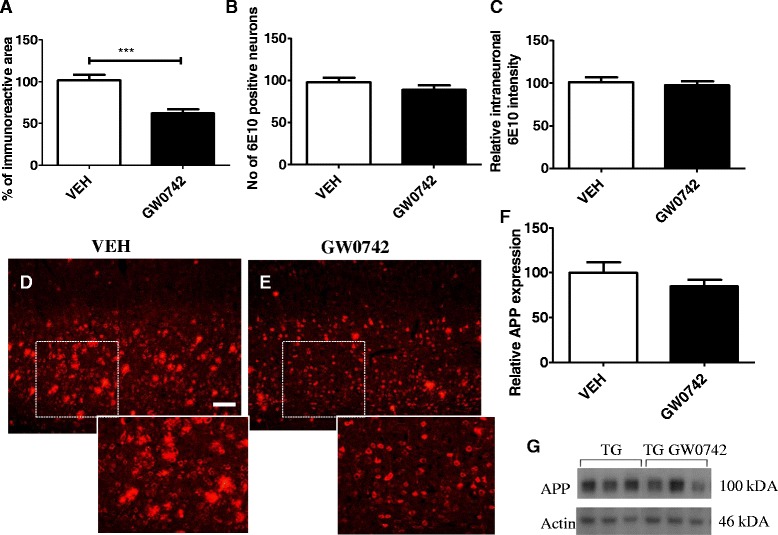
**GW0742 treatment did not reduce the number of neurons with intraneuronal APP/A** **β.** GW0742-treated mice showed less 6E10 immunoreactivity in cortical layer V **(A)** but the number of 6E10 positive neurons in cortical layer V was similar between the vehicle- and GW0742-treated mice **(B)**. The relative intensity of intraneuronal 6E10 immunoreactivity was not altered by GW0742 treatment **(C)**. Figures **(D)** and **(E)** depict typical examples of 6E10 immunoreactivity in cortical layer V of vehicle- **(D)** and GW0742- **(E)** treated mice. GW0742 treatment failed to significantly decrease the levels of brain APP **(F)** as analyzed by Western blotting. Figure **(G)** depicts a typical example image of APP expression levels in TG vehicle- and GW0742-treated mice. Results are presented as mean ± SEM. VEH = vehicle- and GW0742 = GW0742-treated mice. ***P < 0.001 as analyzed by Student’s *t*-test. N = 8 to 9 in vehicle- and n = 9 in GW0742-treated group. Scale bar = 200 μm.

High levels of intraneuronal Aβ/APP accumulate principally in layer V cortical neurons and in the subiculum that are clearly evident as early as 2 months of age [25]. By the age of 5 months, when the mice were sacrificed, the accumulation of intraneuronal Aβ in cortical layer V neurons is significant with a majority of neurons exhibiting 6E10 immunoreactivity within the cell soma. To assess the effect of GW0742 treatment on the extent of Aβ deposition in cortex, we first quantified the levels of layer V total 6E10 immunoreactivity and found that 2 weeks of treatment of 4.5-month-old 5XFAD mice with GW0742 resulted in significant reduction in the levels of 6E10 positive deposits (Figure 3A). To determine whether the GW0742 treatment affected the extent of intraneuronal Aβ accumulation, we then counted the number of 6E10 positive neuronal cell bodies. We found no significant treatment effect on the number of 6E10 positive neurons (Figure 3B). In addition, the treatment did not affect the intensity of intraneuronal 6E10 immunoreactivity (Figure 3C). Since 6E10 detects APP species, and its proteolytic products, we performed Western analysis using 6E10 and quantified the relative APP intensity. The quantification revealed that GW0742 did not significantly reduce the protein levels of full-length APP (Figure 3F).

### GW0742 treatment prevented the loss of NeuN positive neurons in the subiculum

**Figure 4:**
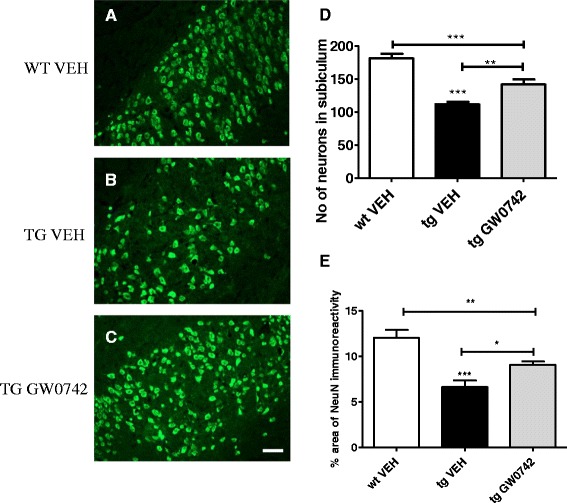
**GW0742 treatment prevented the loss of neurons in the subiculum.** Figures **(A -C)** depict typical example images of NeuN positive neurons in the subiculum of WT vehicle- **(A)**, TG vehicle- **(B)** and GW0742-treated **(C)** mice. NeuN immunopositive cells were manually counted **(D)** and the data were confirmed by quantifying the percentage of NeuN immunoreactive areas in the subiculum of the hippocampi **(E)**. 5XFAD mice exhibited significant loss of NeuN positive cells, which was partially prevented by GW0742 treatment **(D, E)**. Results are presented as mean ± SEM. VEH = vehicle- and GW0742 = GW0742- treated mice. **P < 0.01 ***P < 0.001 as analyzed by 1-way ANOVA followed by Tukey’s *post hoc* test. n = 6 in WT vehicle-treated group, n = 8 in TG vehicle- and n = 7 in TG GW0742-treated group. Scale bar = 200 μm.

The 5XFAD mice exhibit significant loss in the number of NeuN positive neurons in the subiculum between 2 and 6 months of age [25,26]. Quantitation and cell count data confirmed that at the age of 5 months these mice exhibit significantly decreased numbers of NeuN positive neurons and NeuN immunoreactive areas in the subiculum compared to non-transgenic mice (Figure 4). Remarkably, the 2-week treatment with GW0742 between 4.5 and 5 months of age significantly attenuated the loss of NeuN immunoreactivity in the subiculum as measured by both the number of NeuN immunopositive cells as well as quantification of NeuN immunoreactivity (Figure 4). These data provide clear evidence of the neuroprotective effects of PPARδ activation.

### Treatment with GW0742 did not alter the lipidated ApoE levels

**Figure 5:**
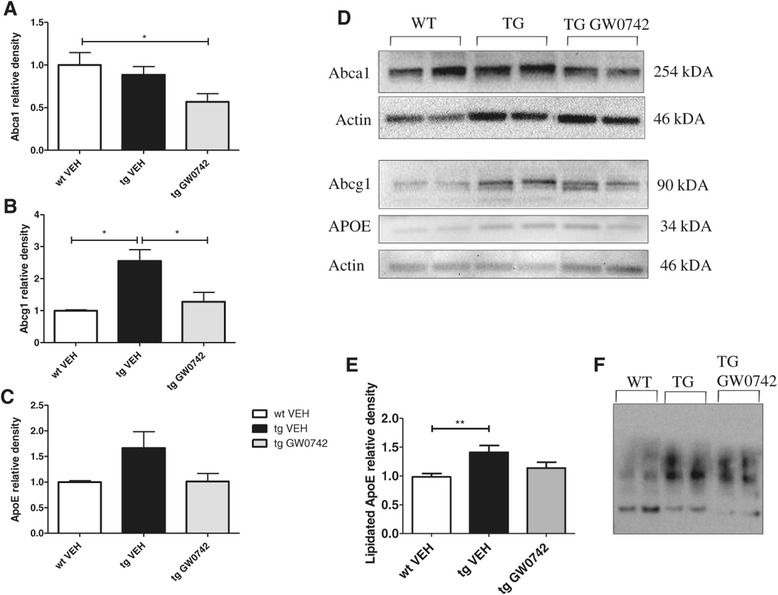
**Treatment with GW0742 did not alter the lipidated ApoE levels.** The protein expression levels of Abca1 **(A)** were unaltered in vehicle-treated TG mice compared to their WT controls whereas the levels of Abcg1 **(B)** were significantly increased. There were no significant changes in the levels of ApoE between the TG vehicle- and WT vehicle-treated mice **(C)**. Two-week treatment with GW0742 slightly reduced the levels of Abca1 **(A)** and the decrease was statistically significant compared to WT vehicle-treated mice but led to significant decrease in Abcg1 compared to TG vehicle-treated mice **(B)**. The levels of ApoE were unaltered by GW0742 **(C)**. Figure **(D)** depicts typical example images of Western blots against Abca1, Abcg1, ApoE and actin as a loading control. TG mice exhibited increased levels of lipidated ApoE in the brain compared to WT mice and the levels were unaltered by GW0742 treatment **(E)**. Figure **(F)** depicts a typical example of the levels of lipidated ApoE **(F)**. Results are presented as mean ± SEM. VEH = vehicle- and GW0742 = GW0742-treated mice. *P < 0.05 **P < 0.01 as analyzed by Student’s *t*-test or 1ne-way ANOVA followed by Tukey’s *post hoc* test. For Abca1, n = 5 in WT vehicle-treated group, n = 6 for TG vehicle-treated group and n = 7 for TG GW0742-treated group. For Abcg1, n = 4 in WT vehicle-treated group, n = 6 for TG vehicle-treated group and n = 8 for TG GW0742-treated group. For ApoE, n = 7 in WT vehicle-treated group, n = 9 in TG vehicle- and n = 11 in TG GW0742-treated group. For ApoE lipidation blot, n = 10 in WT vehicle-treated group, n = 9 for TG vehicle-treated group and n = 9 for TG GW0742-treated group.

Since lipidated ApoE has been shown to be involved in the nuclear receptor-mediated reduction of brain Aβ levels, we analyzed the levels of lipidated ApoE, the total levels of ApoE and the main transporters involved in the ApoE lipidation, Abca1 and Abcg1 from the vehicle and GW0742-treated mouse brain homogenates. Transgenic vehicle-treated mice did not exhibit altered levels of Abca1 compared to WT vehicle-treated mice (Figure 5A); however, the levels in Abcg1 were significantly increased (Figure 5B). The levels of total ApoE were unaltered in vehicle-treated TG mice compared to their WT controls (Figure 5C). Treatment with GW0742 led to slight decrease in the levels of Abca1 reaching statistical significance compared to WT vehicle-treated mice (Figure 5A) and a significant decrease in the levels of Abcg1 compared to the TG vehicle-treated mice (Figure 5B). The treatment had no significant effect on the levels of total ApoE (Figure 5C). The levels of lipidated ApoE were significantly increased in the brain homogenates of TG vehicle-treated mice compared to their WT controls but were unaffected by GW0742 treatment (Figure 5E). These data argue that the salutary effects of PPARδ activation do not arise from regulation of ApoE levels or lipidation status and are thus distinct from the actions of PPARγ and retinoid-X-receptor agonists.

### GW0742-treated mice showed decreased expression levels of brain proinflammatory mediators

**Figure 6:**
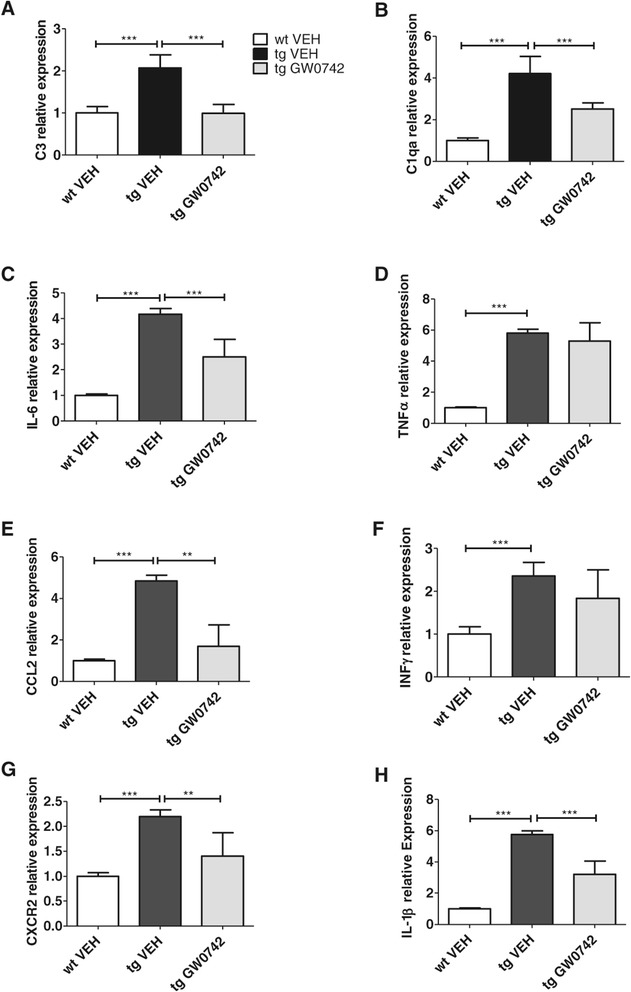
**GW0742 treatment decreased the expression levels of proinflammatory mediators in the brains of 5XFAD mice.** The expression levels of C3 **(A)**, C1qa **(B)**, IL-6 **(C)**,TNFα **(D)**, CCL2 **(E)**, INFγ **(F)**, CXCR2 **(G)** and IL-1β **(H)** in the brain of the 5XFAD mice were analyzed by qPCR. Results are presented as mean ± SEM. VEH = vehicle- and GW0742 = GW0742-treated mice. **P < 0.01 ***P < 0.001 as analyzed by 1-way ANOVA followed by Tukey’s *post hoc* test. n = 10 in WT vehicle-treated group, n = 9 in TG vehicle- and n = 9 in TG GW0742-treated group.

Since GW0742 is known to be an effective anti-inflammatory agent in other tissues, the expression levels of a panel of brain proinflammatory mediators were assessed from the brain homogenates. Quantitative PCR revealed a significant up-regulation in the expression levels of C3 (Figure 6A), C1qa (Figure 6B), interleukin (IL)-6 (Figure 6C), tumor necrosis factor (TNF)α (Figure 6D), chemokine (C-C motif) ligand 2 (CCL2) (Figure 6E), IFNγ (Figure 6F), CXC chemokine receptor 2 (CXCR2) (Figure 6G) and IL-1β (Figure 6H) in the 5XFAD mice compared to non-transgenic controls. Importantly, GW0742 treatment acted broadly to suppress the expression of C3, C1qa, IL-6, CCL2, CXCR2 and IL-1β.

### GW0742-treated mice showed decreased levels of C1qa and C3 immunoreactivity

**Figure 7:**
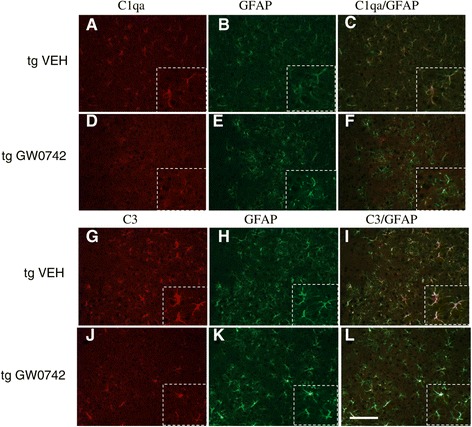
**The expression levels of C1qa and C3 were reduced in GW0742-treated mouse brain as analyzed by immunohistochemistry.** Immunohistochemistry against C1qa and C3 revealed a staining pattern associated predominantly with glial fibrillary acidic protein (GFAP) immunoreactivity. Figures **(A)** and **(B)** show typical example images of C1qa and GFAP immunoreactivities in vehicle-treated TG mice, respectively and figure **(C)** is the overlay of C1qa and GFAP. Figures **(D-F)** show example images of corresponding immunoreactivities in GW0742-treated TG mice. Figures **(G-H)** show C3 and GFAP immunoreactivities in vehicle-treated TG mice and figure **(I)** the overlay of C3 and GFAP. Figure **(J-L)** depict example images of corresponding immunoreactivities in GW0742-treated TG mice. The high magnification insets show typical C1qa and C3 staining pattern resembling astrocytes. Scale bar = 200 μm.

Since the RNA expression levels of C1qa and C3 were decreased in GW0742-treated 5XFAD mice, we confirmed the reduction of C1qa and C3 protein levels by immunohistochemistry. Both C1qa and C3 expression was dramatically reduced upon GW0742 treatment and was mainly co-localized with GFAP immunoreactivity (Figure 7).

### Activation of PPARδ prevented the LPS-induced increase in the expression levels of proinflammatory mediators in primary microglia *in vitro*

**Figure 8:**
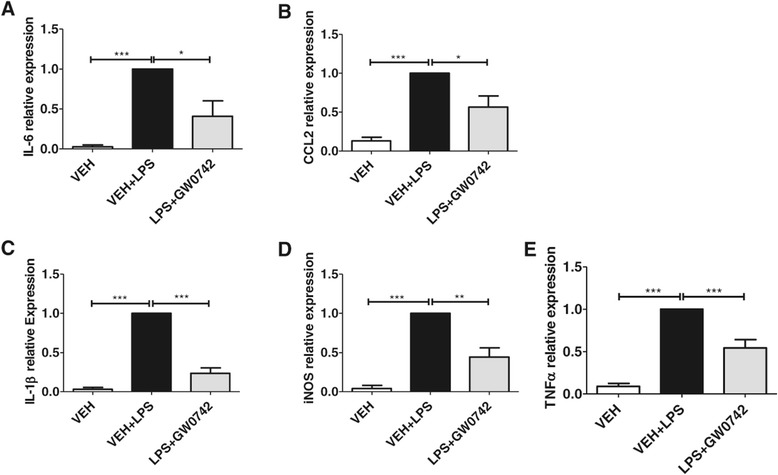
**GW0742 prevented the lipopolysaccharide (LPS)-induced increase in the expression levels of proinflammatory mediators** ***in vitro*** **.** Microglia were exposed to vehicle or GW0742 for 24 hours after which the cells were exposed 10 ng/ml LPS for 24 hours. The expression levels of IL-6, **(A)**, CCL2 **(B)**, IL-1β **(C)**, iNOS **(D)** and TNFα **(E)** were analyzed by qPCR. Results are presented as mean ± SEM. VEH = vehicle- and GW0742 = GW0742-treated mice. **P < 0.01 ***P < 0.001 as analyzed by 1-way ANOVA followed by Tukey’s *post hoc* test. n = 3 to 4 per group derived from individual experiments and values are normalized to VEH + LPS.

To detect whether PPARδ activation shows anti-inflammatory properties *in vitro*, primary microglia were pre-exposed to GW0742 followed by exposure to 10 ng/ml LPS in the presence of GW0742 or vehicle for 24 hours. Analysis of mRNA expression levels by qPCR revealed significant up-regulation in the levels of IL-6 (Figure 8A), CCL2 (Figure 8B), IL-1β (Figure 8C), inducible nitric oxide synthase (iNOS) (Figure 8D) and TNFα (Figure 8E) upon LPS treatment. The levels of all of these proinflammatory mediators were decreased in cultures treated with GW0742 (Figure 8A-E, respectively).

### GW0742 treatment prevented inflammation-mediated neuronal death *in vitro*

We first tested if cultured neurons could be protected from the neurotoxic effects of glutamate by the PPARδ agonist GW0742. Primary neurons were pre-exposed to GW0742 followed by exposure to 500 μM glutamate in the presence of GW0742 or vehicle for 24 hours. There was no significant effect on neuronal survival by GW0742 on glutamate-induced neuronal death. GW0742 alone was not toxic to neurons (data not shown).

**Figure 9:**
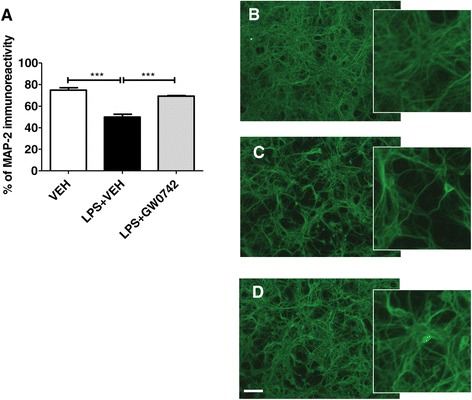
**GW0742 prevented the inflammation-induced neuron death** ***in vitro*** **.** Neurons were cultivated in the presence of microglia and pre-exposed to vehicle or GW0742. Thereafter, the cells were exposed 100 ng/ml LPS/30 ng/ml of IFN-γ for 48 hours in the presence of GW0742 or vehicle and the neuron loss analyzed by quantifying the extent of MAP-2 staining. GW0742 prevented inflammation-induced loss of MAP-2 immunoreactivity **(A)**. Panels **(B-D)** show typical examples of vehicle **(B)**, vehicle + LPS/IFN-γ **(C)** and LPS/IFN-γ + GW0742- **(D)** treated cells. Results are presented as mean ± SEM. VEH = vehicle- and GW0742 = GW0742-treated cells. **P < 0.01 ***P < 0.001 as analyzed by 1-way ANOVA followed by Tukey’s *post hoc* test. n = 3 to 4 (individual experiments) per group. Scale bar = 200 μm.

Microglia elaborate an array of cytotoxic products that are postulated to contribute to neuronal death in AD. We next employed tissue culture to assess whether GW0742 protects neurons against inflammation-induced neuronal degeneration in neuron-microglia co-cultures. Primary neurons were cultivated in the presence of primary microglia and pre-exposed to GW0742 and, thereafter, exposed to 100 ng/ml LPS and 30 μg/ml IFNγ for 48 hours in the presence of GW0742 or vehicle. In the co-culture system neuronal death cannot be assessed by MTT assay. Therefore, the effect of GW0742 on neuronal survival against inflammation-induced neuron loss was monitored by MAP-2 staining. Quantification of MAP-2 immunoreactivity revealed significant loss of MAP-2 immunoreactive area in vehicle- treated cells compared to GW0742-treated cells (Figure 9).

## Discussion

Here we report that PPARδ activation in an animal model of AD results in reduction in the extracellular plaque burden that is associated with a robust reduction in inflammation. Importantly, treatment with a PPARδ agonist provided robust neuroprotection, with a significant attenuation of the loss of neurons in the subiculum of 5XFAD mice. Neuronal death is one of the main pathological features in patients with AD. The current study is thus far the only study linking PPARδ-mediated reduction in the Aβ load with preservation of neurons in a transgenic mouse model of AD. Importantly, our study shows that the protection of the neurons was not associated with a reduction in intraneuronal Aβ by PPARδ activation, but rather with a suppression of inflammation. Of importance is that a significant reduction in the levels of plaque-associated 6E10 immunoreactivity was achieved with a brief 2-week treatment period. The fact that the amount of Aβ-containing neurons and their 6E10 immunoreactivity was not altered upon GW0742 treatment suggest that our GW0742 treatment paradigm had a direct effect on microglia and did not affect the accumulation of intraneuronal Aβ.

An earlier study showed that PPARδ activation led to decreased levels of 6E10 immunoreactivity only in the subiculum of 5XFAD mice [18]. This is in accordance with our data showing decreased levels of 6E10 immunoreactivity in the subiculum, but in addition we also noted a significant decrease in the levels of Aβ deposits in hippocampus and in cortical layer V. The reduction was specific to extracellular deposits since the number of 6E10 positive neurons remained unchanged upon treatment. There are three major differences between the current study and the study by Kalinin *et al*. First, the age of the animals at the start of the treatment in our study was 4.5 months compared to 2 or 3.5-month-old mice used in the study by Kalinin *et al*. The second is the treatment time, which was only 2-weeks in the current study compared to 1-month period in the study by Kalinin *et al*. Lastly, we administered the drug by oral gavage once a day, whereas Kalinin *et al.* provided the drug in chow. Our study shows that PPARδ agonist GW0742 is very effective in reducing the levels of extracellular Aβ deposits over a relatively short treatment period. In contrast to Kalinin *et al*., the mechanism underlying the clearance of Aβ was not mediated through increased neprilysin (NEP) or insulin-degrading enzyme (IDE) expression, as we were unable to detect GW0742-stimulated mRNA expression levels of NEP and IDE (data not shown). Instead, we postulate that the reduction is due to enhanced microglial-mediated clearance of Aβ.

A large body of evidence suggests that ApoE is one of the main mechanisms underlying soluble Aβ clearance within the interstitial fluid in the AD brain [9,10] and is involved in microglia-mediated degradation of soluble Aβ [8-11,27]. Since some studies have suggested that PPARδ agonists induce the expression of Abca1 [28,29], we wanted to analyze whether the reduction in the Aβ deposition was due to increased expression of Abca1 or Abcg1 and subsequent increase in ApoE lipidation. Our results show that GW0742 treatment unexpectedly reduced the protein expression levels of these proteins. Whilst this may be attributed to differences in the models used in previous studies [28,29], it is important to note that the levels of total ApoE and lipidated ApoE were not altered by GW0742 suggesting that the effect of GW0742 is not mediated through induction of ApoE lipidation. This is consistent with published literature as no data showing elevation in the levels of ApoE upon PPARδ activation have been reported to date in this context, nor are predicted from the sequence of the nuclear receptor response element in the ApoE promoter.

In the current study GW0742 treatment reduced the expression levels of several cytokines both *in vitro* in primary microglia and *in vivo* in TG mouse brain. This strongly supports the hypothesis that PPARδ treatment leads to reduced cytokine expression profile in multiple proinflammatory cytokines, which has beneficial effects on microglial activation and induces concomitant reduction in the brain Aβ burden. The fact that we detected similar changes both in primary microglia *in vitro* and in 5XFAD mouse brain supports the contribution of microglia in the observed effects. A large number of cytokines and their corresponding receptors have been shown to be elevated in AD brain and the increased levels of many, such as IL-1β have detrimental effects on neuronal survival [30]. CCL2 and CXCR2 are amongst inflammatory mediators the levels of which have been shown to be linked to neurodegeneration [31-34]. Similar to our study, different treatment paradigms in animal models of AD have demonstrated the link between reduction in the levels of CCL2 or CXCR2 and decreases in brain Aβ [35-38]. The fact that GW0742 induced a significant reduction in several measured cytokines implies that the potency of the drug relies on reducing the inflammatory milieu and inducing the concomitant beneficial actions in AD-like pathology.

In the current study the expression levels of C1qa and C3 were significantly elevated in the brains of 5XFAD mice and were reduced by GW0742 treatment. Most of the C1qa and C3 immunoreactivity was associated with GFAP immunoreactivity suggesting that although the level of astrocytic activation was not reduced, astrocytic expression of C1qa and C3 was diminished. The human AD brain has been shown to have increased expression of complement proteins [39]. Aβ has been shown to directly activate the complement pathway [40-43] prompting the hypothesis that the activation of the complement pathway is detrimental in AD. Whereas microglial expression of complement proteins C1qa and C3 may enhance the Aβ phagocytic capacity [44-46], increased expression of these proteins has also been shown to lead to neuronal degeneration and death [47-49]. The overall decrease in C3 and C1qa in the current study may reflect a decrease in the brain inflammatory milieu. This may be beneficial for neuronal survival.

An important finding in our study is that treatment of AD mice with GW0742 not only reduced the brain inflammatory milieu but also prevented the loss in NeuN immunoreactivity in a specific brain area in 5XFAD mice that is susceptible to robust neuronal death [25,26]. We confirmed this finding *in vitro* by using primary neuron-microglia co-cultures where GW0742 preserved MAP-2 immunoreactivity against inflammation-induced neuronal death. Although NeuN may not be a confirmative marker for neuronal death [50] and our data do not represent absolute neuronal counts, our data are supported by studies showing the neuroprotective effect of PPARδ agonists in various models of neurodegenerative diseases [13,15-17,51] mainly via reducing inflammation and oxidative stress. It is noteworthy that GW0742 alone was not neuroprotective against glutamate exposure at the concentrations used in this study. The ability of GW0742 to provide direct neuroprotection *in vitro* has yielded some contradictory results and may vary depending on treatment times, exposure concentrations and cellular models used and direct neuroprotection may require very high concentrations of PPARδ agonists [13,52,53]. Our data imply that rather than providing direct neuroprotection against glutamate-induced neuronal death, GW0742 protects primary neurons from inflammation-induced neuronal death at the concentrations of GW0742 used in the study.

Our study shows for the first time that enhancement of PPARδ activity with a relatively short time window of only 2 weeks resulted in significant decrease in the amount of Aβ deposits and, importantly, it induced an overall decrease in the proinflammatory milieu slowing down the neuronal deterioration of 5XFAD mice. Our data warrant the activation of PPARδ as a potential therapeutic strategy to combat AD.

## Abbreviations

AD: Alzheimer’s disease
PPAR: peroxisome proliferator activator receptor
Aβ: beta-amyloid
PD: Parkinson’s disease
APP: amyloid precursor protein
WT: wild type
TG: transgenic
GFAP: glial fibrillary acidic protein
CTF: C-terminal fragment
Iba-1: ionized calcium binding adaptor molecule 1
VEH: vehicle
ApoE: apolipoprotein E
Abca1: ATP-binding cassette transporter A1
Abcg1: ATP-binding cassette transporter G1
C3: complement component 3
IL: interleukin
TNFα: tumor necrosis factor α
CCL2: chemokine (C-C motif) ligand 2
IFNγ: interferon γ
CXCR2: CXC chemokine receptor 2
LPS: lipopolysaccharide
iNOS: inducible nitric oxide synthase
NEP: neprilysin
IDE: insulin-degrading enzyme
MAP-2: microtubule-associated protein 2
